# Microbiology and Clinical Outcome of Hospital-Acquired Respiratory Infections in an Italian Teaching Hospital: A Retrospective Study

**DOI:** 10.3390/healthcare10112271

**Published:** 2022-11-12

**Authors:** Massimo Maurici, Gian Loreto D’Alò, Carla Fontana, Viviana Santoro, Roberta Gaziano, Marco Ciotti, Domenico Cicciarella Modica, Patrizia De Filippis, Loredana Sarmati, Gerardo De Carolis, Francesca Pica

**Affiliations:** 1 Department of Biomedicine and Prevention, University of Rome “Tor Vergata”, 00133 Rome, Italy; 2 IRCCS Istituto Nazionale per le Malattie Infettive “Lazzaro Spallanzani” (INMI), Via Portuense 292, 00149 Rome, Italy; 3 Department of Experimental Medicine, University of Rome “Tor Vergata”, 00133 Rome, Italy; 4 Laboratory of Microbiology and Virology, Fondazione Policlinico Tor Vergata, 00133 Rome, Italy; 5 Department of System Medicine, University of Rome “Tor Vergata”, 00133 Rome, Italy; 6 Hospital Health Direction, Fondazione Policlinico Tor Vergata, 00133 Rome, Italy

**Keywords:** hospital-acquired respiratory infection (HARI), microorganisms, antimicrobials, hospitalized patients, clinical outcome

## Abstract

The burden, microbial etiology and clinical impact of hospital-acquired respiratory infections (HARIs) were determined at an Italian teaching hospital over a 12-month period. For this purpose, overall ordinary hospitalizations ≥ 2 days of subjects over 18 years old with discharge from 1 January 2018 to 31 December 2018 were examined by cross-referencing demographic and clinical data from hospital discharge forms with microbiological data from the computer system of the Microbiology Unit. We identified 329 individuals with HARIs (96 females and 233 males; median age 70 years, range 18–93), who represented ¼ of the total hospital-acquired infections (HAIs) in the period. The inpatient setting was medical and surgical in similar proportions (169 vs. 160, respectively) and the mean hospital stay was 38.9 ± 33.6 days. One hundred and forty patients (42.6% of the total sample) were suffering from one or more chronic diseases. A total of 581 microorganisms (82 antibiotic-resistant and 499 non-resistant) were detected in HARI patients. The most common isolated species were *Staphylococcus aureus* (16.7%), *Klebsiella pneumoniae* (13.3%), *Pseudomonas* spp. (12.6%) and *Acinetobacter baumannii* (10.5%), followed by *Enterobacter* spp. (5.3%), *Escherichia coli* (5.2%) and *Enterococcus* spp. (4.8%). One hundred and sixty-seven individuals (49.0% of the total) had polymicrobial infections. One hundred thirty-one patients (39.8% of the total) underwent endotracheal intubation and mechanical ventilation and 62.6% of them died, compared to 17.7% of the non-intubated patients. Multivariable analysis confirmed a positive correlation between death and increased age (*p* = 0.05), surgical MDC (*p* = 0.007), number of microorganisms over the sample mean (*p* = 0.001), the presence of chronic diseases (*p* = 0.046), and intubation and mechanical ventilation (*p* < 0.0001). A positive correlation between intubation and antibiotic-resistant organisms (*p* = 0.003) was also found. HARIs are still a major public health problem and require constant surveillance due to their severe clinical outcome.

## 1. Introduction

Hospital-acquired infections (HAIs) appear 48 h or more after hospital admission [1]. Although some of these infections can be easily treated, others can affect patients’ health more severely, often leading to poor prognosis. In recent years, international scientific research has focused on the prevention and control of HAIs, due to their growing epidemiological trend and their significant impact on the health of hospitalized patients, as well as on the financial aspects of health management because of the prolongation of the length of hospitalization, long-term disability, increased mortality, and the spread of antibiotic resistance [2]. The expansion of HAIs is influenced by various factors, such as the increased prevalence of frailty in the population, the spread of microorganisms resistant to antimicrobial drugs, and the progressive introduction of new health technologies that guarantee the survival of patients in serious conditions but also allow the entry of microorganisms into normally sterile body sites [3]. The most frequently reported HAIs affect the respiratory tract, surgical site, urinary tract, bloodstream and gastro-intestinal tract [4].

In the present report, we focused on hospital-acquired respiratory infections (HARIs) that are common in hospitalized patient populations and that threaten morbidity and mortality [5,6,7]. HARIs can be caused by bacterial, viral, or fungal agents, depending on patient exposure and clinical risk factors. Bacterial pathogens associated with HARIs, including *Staphylococcus aureus*, *Pseudomonas aeruginosa*, *Acinetobacter baumannii*, *Klebsiella pneumoniae* and members of the *Enterobacterales*, are often multidrug-resistant [2]. This includes carbapenemase-producing organisms, which have been independently associated with high mortality rates following infection [8].

HARIs include upper and lower respiratory tract infections, both of which can significantly impact the clinical outcome of hospitalized patients. The etiological diagnosis of HARIs is not made in all cases, but is most common in clinically severe cases and in patients admitted to intensive care units (ICUs), as demonstrated in a recent study [9]. HARIs include different clinical entities, but the most relevant are hospital-acquired pneumonia (HAP) and ventilator-associated pneumonia (VAP) [10,11]. While HAP is independent from mechanical ventilation, VAP is diagnosed in patients who have been mechanically ventilated for ≥48 h [12]. It is possible, however, to find some HAP patients requiring mechanical ventilation to treat respiratory failure (v-HAP), which is characterized by an increased clinical severity (due to respiratory failure) compared to non-ventilated HAP (nv-HAP) [12].

It is known that the epidemiology and antimicrobial profiles of the microorganisms involved in HARIs vary over time, not only among different institutions, but also within the same hospital wards. Therefore, every hospital center should be familiar with its local microbiological trends in order to adopt the most appropriate empirical therapy and preventive strategies [13,14].

In order to improve the knowledge of the epidemiological situation at Tor Vergata Teaching Hospital, we retrospectively analyzed the demographic, clinical and microbiological data (available on separate databases) of patients with documented HARIs and their discharge flows in a 12-month period. This study approach represents a useful tool for a fast and accurate characterization of the epidemiology of HAIs and for the implementation of more adequate preventive and therapeutic interventions.

## 2. Materials and Methods

### 2.1. Study Design

This retrospective observational study was conducted at the Teaching Hospital Policlinico Tor Vergata (PTV), Rome, Italy. The study focused on the microbiological and clinical aspects of HARIs observed over a 12-month period. To this end, the overall ordinary hospitalization data at the PTV for discharge from 1 January 2018 to 31 December 2018 for medical and surgical wards have been evaluated. Data extraction was carried out by analyzing hospital discharge records (HDRs) using the computerized hospital information system (HIS) database. These data (i.e., diagnoses and procedures) have been compiled by means of the AREAS ADT (admission, discharge and transfer) information system, using the classification of ICD-9-CM codes (2007 version). The layout thus composed was further processed for the evaluation of quantitative/qualitative variables, as well as MDC (major diagnostic category)—which identifies medical and surgical patients—obtained from the diagnosis-related groups (DRG) grouper ver. 24.0. The reports of the microbiological analysis carried out on the respiratory samples sent by the various PTV wards to the microbiology lab from 1 January to 31 December 2018 were extracted from the computer system of the microbiology unit of the PTV, together with the relative collection dates.

### 2.2. Record Linkage

The binding work involved, first, the part cleaning of the databases, which concerned individual fields of personal data (names of the patients and dates of birth), to proceed to a semi-deterministic agreement between the databases of the laboratory. For this purpose, the types of clinical specimens (bronchoalveolar lavage fluid (BAL), bronchial aspirate (BAS), adequate sputum specimen, or tracheal swab) were used as keys, in addition to the personal data, in order to identify a suspected HARI. Subsequently, based on the nosological number used as a deterministic key, a unique database was created showing the flow of HDR 2018, the first admissions, and the collection of clinical samples based on the type of infection under study, i.e., respiratory infections. 

### 2.3. Inclusion and Exclusion Criteria

The ordinary hospitalizations (HDR code 1 variable “type of hospitalization”) of all the hospital wards were included in the analysis of patients with a discharge on 31 December 2018. Non-ordinary admissions were excluded (HDR code 2—day hospital; code 3—home treatment; code 4—day surgery with overnight stay). Age <18 years was a further exclusion criterion. Infections that occurred in the first 48 h of hospitalization were excluded from the analysis, as per the definition of HAI. For each microorganism detected during the hospitalization, the dates of the first and last detection before discharge were included.

### 2.4. Considered Variables

The following variables were collected from the HDRs: personal information (age and gender); educational level; nationality; marital status; occupation; length of hospital stay; type of discharge; primary diagnosis and secondary diagnoses (from 1st to 5th) at discharge; and codes relating to the procedures performed during hospitalization (all procedures were coded with ICD-9-CM ver. 2007). MDCs comprise 25 mutually exclusive categories into which all possible principal diagnoses fall. The diagnoses in each category correspond to a single body system or etiology, broadly reflecting the specialty care provided. Each MDC was partitioned according to whether or not a surgical procedure was performed. This preliminary partitioning into MDCs occurred before a diagnosis-related group was assigned.

We identified intubated patients by the following ICD-9-CM (ver. 2007) codes: 31.1—temporary tracheostomy; 31.29—other permanent tracheostomy; 96.04—insertion of endotracheal tube; 96.70—continuous mechanical ventilation of unspecified duration; 96.71—continuous invasive mechanical ventilation for less than 96 consecutive hours; 96.72—continuous invasive mechanical ventilation for 96 consecutive hours or more.

The education level was divided into two separate categories, i.e., below and equal/over high school. Below high school was designated to subjects who attended primary and lower secondary school, while equal/over high school was designated to subjects who graduated high school, attended college and received bachelor’s, master’s, or doctoral degree.

The following information was extracted from the microbiology lab database: positive sampling for microorganisms with relative sampling dates and requests from the sending department; type of clinical sample collected; and positivity of the antimicrobial resistance phenotype tested. All data were processed by creating index variables to perform statistical analysis.

### 2.5. Microorganism Identification

After cultivation on traditional media, microorganisms were identified using matrix-assisted laser desorption/ionization time-of-flight mass spectrometry (MALDI-TOF MS; Brucker Daltonics), as previously described [15]. Antimicrobial susceptibility testing was performed using Micronaut panels (Diagnostika Gmbh, Bornheim, Germany, now company of Bruker Daltonics, Billerica, MA, USA) run on MICRO MIB (Bruker Daltonics, Billerica, MA, USA) and interpreted following the European Committee on Antimicrobial Susceptibility Testing (EUCAST) clinical breakpoint (v 8.1_2018). The identification of carbapenemases (KPC, VIM, IMP, NDM, and OXA48) was performed using the immunochromatographic assay NG CARBA (NG Biotech, Guipry-Messac, France) according to the manufacturer’s instructions, and then confirmed by PCR assay [16]. The antibiotic resistance profiles were determined according to the definitions reported by Magiorakos et al. [17]: multidrug-resistant (MDR) bacteria are non-susceptible to ≥1 agent in ≥3 antimicrobial categories; extensively drug-resistant (XDR) bacteria are non-susceptible to ≥1 agent in all but ≥2 categories; pandrug-resistant bacteria (PDR) are non-susceptible to all antimicrobial agents listed [17]. The lists of antimicrobial categories and agents used for the above definitions are specific for each individual microorganism and are based on the recommendations included in EUCAST Expert Rules v 3.2 (February 2020) http://www.eucast.org/expert_rules_and_intrinsic_resistance/ (accessed on 29 October 2022).

### 2.6. Ethics Statement

The study was performed according to the Declaration of Helsinki and in accordance with the International Conference on Harmonization Good Clinical Practice Guidelines (ICH-GCP E6 (R2)). The Independent Ethical Committee of the University of Rome “Tor Vergata” approved the study protocol (Approval No. 216/19, 18 December 2019).

### 2.7. Statistical Analysis

Continuous variables (age and length of hospital stay) were expressed in terms of mean and standard deviation, median, and IQR (including minimum and maximum values) where appropriate. All the other variables were represented in terms of absolute numbers and percentage. We chose to categorize all the non-continuous variables into dichotomous ones. To assess the normality of the continuous variables we performed the Shapiro–Wilk test and observed that normality assumption should be rejected for all considered variables. Thus, we chose to perform the Mann–Whitney U test to compare the means of both age and length of stay.

Descriptive analyses of the sample obtained following record linkage, risk analysis (odds ratio and Pearson chi-square), and the Mann–Whitney U test were conducted to observe the statistical significance of the associations investigated.

The isolated microorganisms (bacteria and fungi) have been summarized by species, in terms of absolute number and percentage with respect to the subgroup considered (antibiotic-resistant, non-resistant, and total). It should be specified that, in our study, the documented resistance of a microorganism to even a single antimicrobial drug led to its inclusion in the group of antibiotic-resistant microorganisms. This definition was the one used by the alert system in function within the hospital and we used the above-mentioned “antibiotic-resistant” classification to perform inferential analyses.

In relation to the dependent variables “death” and “ventilation/intubation”, we performed logistic regression analysis, including in the model the independent variables that were found to be associated at the univariable level with a *p* value ≤ 0.10. The statistical significance for the logistic regression model was set at *p* ≤ 0.05. We also tested independent variables for multicollinearity, removing, if necessary, those with a Pearson correlation coefficient > 0.3. No variables needed to be removed because all VIF (variance inflation factor) values were <0.3. We performed the Hosmer–Lemeshow (HL) goodness-of-fit (GOF) test and calculated Nagelkerke R2 values to assess both the quality of the multivariable model and the fitness of the predictive model.

In relation to the dependent variable of “length of hospital stay”, we performed a linear regression, testing all the relevant assumptions, and including all the socio-demographic and clinical independent variables in the multivariable model. The significance for the linear regression model was set at *p* ≤ 0.05.

Statistical analysis was performed with SPSS software v.22.0 (SPSS Inc., Chicago, IL, USA).

## 3. Results

### 3.1. Study Population

The patients hospitalized for ≥2 days at PTV in the observation period were 11,166 (91.4% of the total hospitalizations in the year); 1352/11,166 (12.1%) were found to have acquired at least one HAI during the observation period, and among these 329/1352 (24.3%) had an HARI. Therefore, HARIs accounted for 2.94% of the total hospitalizations ≥ 2 days.

Demographic and clinical characteristics of the 329 patients with documented HARIs are shown in Table 1.

Among them, there were 96 female and 233 male individuals. A total of 128 individuals were <65 years of age, whereas the remaining 201 were 65 years old or older. Although in the studied population there were more males than females, there were no significant differences in the average age compared to gender (data not shown). Almost all of the patients were Italians with a high level of education, mostly married and retired or unemployed (Table 1). The inpatient setting was medical or surgical in almost equal proportions. The mean hospital stay was 38.9 days (Table 1).

One hundred and forty patients (42.6% of the total sample) were suffering from one or more chronic diseases (Table 1). The distribution of chronic diseases among the studied patients was as follows: lung disease (*n* = 46, 14.0%), heart failure (*n* = 45, 13.7%), kidney failure (*n* = 51, 15.5%) and onco-hematological (*n* = 24, 7.3%) diseases. Mortality rate according to different chronic diseases among the 117 dead patients was: lung disease (*n*= 11, 9.4% of the total), heart failure (*n* = 22, 18.8%), kidney failure (*n* = 35, 29.9%) and onco-hematological diseases (*n* = 6, 5.1%).

One hundred and thirty-one patients (39.8% of the total sample) underwent endotracheal intubation and/or invasive mechanical ventilation (Table 1). There was a higher percentage of patients with resistant organisms among those who had been intubated and mechanically ventilated (55/131; 42.0%) than in those who had not undergone this procedure (20/198; 10.1%), with an odds ratio (OR) of 6.44 [CI 95% 3.61 to 11.48, *p* < 0.0001].

Overall, 117 patients (35.6% of the total sample) died during hospitalization. Eighty-two out of the 117 dead patients (70.1%) were patients subjected to endotracheal intubation and invasive mechanical ventilation, while the remaining 35 (29.9%) did not undergo this intervention. Therefore, mortality in intubated and mechanically ventilated patients was 62.6% (82/131) vs. 17.7% (35/198) in non-ventilated patients.

Overall, 75 out of 329 patients (22.8% of the population under study) tested positive for the presence of antibiotic-resistant organisms, and 7 of them had more than one resistant organism (Table 1). Amongst the 75 patients with resistant organisms, 41 deaths (54.7%) were recorded: 38 out of the 55 patients (69.1%) who underwent intubation and mechanical ventilation and 3 out of the 20 patients (15%) who did not receive this treatment [OR 12.66, CI 95% 3.27 to 49.06, *p* = 0.00003]. Among the 254 patients with non-resistant organisms, 76 deaths (29.9%) were recorded.

### 3.2. Microorganisms

A total of 581 microorganisms were detected in the respiratory samples from the 329 patients with HARI. The respiratory samples analyzed included bronchoalveolar lavage fluid, bronchial aspirate, adequate sputum specimen and tracheal swab. Microbiological analysis of the isolated organisms showed that 57.1% (*n* = 332) of them were Gram-negative bacteria, 24.1% (*n* = 140) Gram-positive bacteria, 0.3% (*n* = 2) *Mycobacterium tuberculosis* and 18.4% (*n* = 107) fungal isolates.

The distribution of microbiological isolates from the respiratory samples of the 329 HARI patients is reported in Table 2, where the percentage values for each microorganism are calculated based on the total of the specific field (i.e., resistant, non-resistant and total number). By analyzing the isolated microorganisms per group, we found that in the Gram-positive group, *S. aureus* (69.8%) and *Enterococcus* spp. (19.4%) were the prevalent bacteria, followed by *Streptococcus pneumoniae* (5.8%), *Streptococcus pyogenes* (4.3%) and *Rothia* (0.7%). In the Gram-negative group, *Klebsiella* spp. (23.1%), *Pseudomonas* spp. (21.6%) and *A. baumannii* (18.6%) were the most prevalent microorganisms, followed by *Enterobacter* spp. (9.3%), *Escherichia coli* (9.0%), *Stenotrophomonas maltophilia* (4.5%), *Proteus* spp. (3.9%) and others. All but three *Pseudomonas* spp., namely, *P. monteilii*, *P. jinjuensis* and *P. putida*, were identified as *P. aeruginosa*. *Candida* spp. accounted for the majority of fungal isolates (87.0%), followed by *Aspergillus* spp. (12.1%). Among the 92 identified *Candida* spp., 63 were *C. albicans*, 10 *C. tropicalis*, 8 *C. glabrata*, 4 *C. krusei*, 3 *C. parapsilosis*, 2 *C. lusitaniae*, 1 *C. dubliniensis*, and 1 *C. kefyr*. One hundred and sixty-seven patients (49% of the total) had a number of microorganisms over the sample mean (i.e., ≥2) in their respiratory secretions, indicating the presence of polymicrobial infection (Table 1). As shown in Table 2, 82 out of the 581 isolated microorganisms were antibiotic-resistant. Taken together, *A. baumannii*, *K. pneumoniae* and *S. aureus* accounted for the majority (62.2%) of antibiotic-resistant organisms. Considering the antimicrobial resistance profile in relation to the single strains and using the definition of Magiorakos and coll. [17], we identified 22 MDR and 2 PDR *A. baumannii*, 9 PDR and 5 MDR *K. pneumoniae* and 13 MDR *S. aureus*. No *Pseudomonas* spp. isolates exhibited antibiotic resistance (Table 2). Six *Aspergillus* spp. isolates were found to be azole-resistant, whereas all *Candida* spp. isolates were non-resistant (Table 2).

### 3.3. Univariable and Multivariable Analysis

The crude and adjusted ORs (and 95% CI) for variables potentially associated with death, and intubation/ventilation are shown in Table 3 and Table 4, respectively.

Considering death as the dependent variable in univariable analysis, we found that some variables showed a *p* value ≤ 0.10 (Table 3). They were: age, marital status, MDC, number of isolated microorganisms over the sample mean (1.76 ± 0.96), the presence of antibiotic-resistant microorganisms, the presence of chronic diseases, and intubation and mechanical ventilation. In contrast, gender, length of hospitalization, citizenship, education level and work activity showed a *p* value > 0.10 (Table 3). Multivariable analysis confirmed the existence of a positive correlation of death with increased age (*p* = 0.05), surgical MDC (*p* = 0.007), number of microorganisms over the sample mean (*p* = 0.001), the presence of chronic diseases (*p* = 0.046), and intubation and mechanical ventilation (*p* < 0.0001). Only marital status (*p* = 0.473) and the presence of antibiotic-resistant microorganisms (*p* = 0.558) did not show a significant correlation with the dependent variable (Table 3).

Considering intubation/ventilation as the dependent variable, the variables found to be significant in univariable analysis were: length of stay (*p* = 0.0001), death (*p* = 0.000), male gender (*p* = 0.043), surgical MDC (*p* = 0.0001), number of microorganisms over the sample mean (*p* = 0.0001) and the presence of antibiotic-resistant microorganisms (*p* = 0.001). However, multivariable analysis confirmed the presence of a positive correlation between intubation/ventilation and death (*p* = 0.0001), male gender (*p* = 0.043), surgical MDC (*p* = 0.0001) and presence of antibiotic-resistant organisms (*p* = 0.003) (Table 4).

Finally, by using a linear regression model, we found a statistically significant correlation between the average hospital stay and death (*p* = 0.0002), surgical MDC (*p* = 0.0001), patients with at least one antibiotic-resistant microorganism (*p* = 0.015), patients with a number of microorganisms over the sample mean (*p* = 0.0003), and ventilation/intubation (*p* = 0.022). Regarding the impact on length of stay, the statistically significant variables were associated with the following changes in terms of days: death: −11.1 days; surgical MDC: +14.2 days; patients with antibiotic-resistant microorganisms: +7.8 days; patients with a number of microorganisms over the sample mean: +9.5 days; ventilation/intubation: +7.5 days (Table 5). 

## 4. Discussion

Despite control efforts, the burden of healthcare-associated infections (HAIs) is high worldwide and in Europe leads to around 37,000 deaths each year [18]. About 8.9 million cases of HAIs per year have been estimated, among which 1/3 are caused by antibiotic-resistant organisms [2]. A recent Italian time prevalence study documented an 8.03% prevalence of patients with at least one HAI and indicated respiratory tract infections as the most frequent HAIs in hospital settings (23.4%) [19].

In our study, an overall HAI incidence of 12.1% was found, which is consistent although slightly higher than the above-mentioned reports. In this regard, it should be considered that the results of studies based on the calculation of the average HAI prevalence vary according to multiple factors, i.e., size of the hospital, type of hospital ward, age of patients, severity of clinical conditions, exposure to invasive devices [18] and complexity of interventions. Moreover, it is known that epidemiological trends vary significantly over time in the same institution; therefore, there is a need for a timely update of HAI incidence and of the circulating microbial species in hospitals, as well as in other healthcare facilities. It is interesting to note that, in our retrospective analysis, HARIs accounted for 24.3% of the total number of HAIs in the period examined, in perfect agreement with the values reported in the studies mentioned and above [2,18,19].

The majority of our patients with HARI were aged 65 years or older and 42.6% of them suffered from one or more chronic diseases, a figure that once again confirms age and comorbidities as two leading factors in increasing the risk to be hospitalized and to develop severe and often life-threatening infections, as reported previously [20]. Consistently, 117 out of 329 patients with HARI (35.6%) died during hospitalization and 82 of them (70.1%) had undergone intubation and mechanical ventilation. Thus, invasive mechanical ventilation is confirmed, even in our study, as related to poor prognosis. Pneumonia associated with mechanical ventilation is a subset of hospital-acquired pneumonia [6,7]. The most common cause of VAP is the microinhalation of bacteria that colonize the oropharynx and upper airways in severely ill patients. Endotracheal intubation is the main risk factor for pneumonia associated with mechanical ventilation, as it affects the airway defenses, reduces cough and mucociliary clearance, and facilitates the microinhalation of bacteria-laden secretions that stagnate above the inflated balloon of the endotracheal tube. Furthermore, the bacteria form biofilms on the endotracheal tube, which protect them from antibiotics and host defenses. In our population sample, the variable of intubation/mechanical ventilation was found to be positively correlated not only with death, but also with male gender, surgical MDC, and presence of antibiotic-resistant organisms (Table 4). This is not surprising when considering that males and surgical patients have a greater tendency to develop localized as well as systemic infections, sometimes severely, and that it is common to observe that males are more often admitted to intensive care units [21,22].

In the respiratory samples from our HARI patients, 581 microorganisms were identified, equal to an average of 1.76 ± 0.96 organisms per patient (range 1–5). The identified microbial species were similar to those reported in other studies [11,23,24,25,26,27,28], with the only exceptions of the two *M. tuberculosis* species, which most likely represented pre-existing co-infectants in our population sample. We also detected polymicrobial infections—in our sample, defined as the presence of microorganisms above the sample mean, i.e., ≥2—in 49% of patients, with a prevalence similar to previous studies on HARIs [29]. This condition has important implications for patient management, as it modifies the selection of antimicrobial therapy and the anticipated response to treatment, thus changing the clinical course of the disease [30,31]. Consistently, in our study, polymicrobial infections were associated with both increased length of stay (Table 5) and higher risk of death (Table 3), as also reported in the literature [32,33,34]. Moreover, antibiotic resistance was found to be correlated with increased length of stay (Table 5) in the multivariable analysis; this finding is consistent with data reported in the literature [35,36], showing that antibiotic resistance leads to additional hospital costs estimated between 23.8% and 29.3% [36].

As expected, among the isolated organisms, there were more Gram-negative than Gram-positive bacteria. In the Gram-negative group, *A. baumannii*, *P. aeruginosa* and *K. pneumoniae* were the most represented. It is known that *Acinetobacter* and *Pseudomonas* are common causes of infections associated with hospitalization [37] and that the most prominent infection caused by these pathogens is VAP [38]. However, the environmental distribution of *Pseudomonas* and *Acinetobacter* is only partially overlapping. In fact, *P. aeruginosa* prefers humid environments, such as water, soil and plants, and it is rarely part of the microbiota of healthy people. *Acinetobacter* can survive on both moist and dry surfaces, can be a normal inhabitant of human skin, and may occasionally be a contaminant of blood cultures [39]. Therefore, in hospital settings they may be passed from person to person (presumably from the hands of healthcare workers) or via environmental contamination [37]. Moreover, for *K. pneumoniae*, a well-known life-threatening human pathogen associated with hospital and community infections, including respiratory and urinary tract infections, blood infections and liver abscesses, there is increasing evidence that infections are often acquired in a hospital setting and develop mainly in patients with impaired immune response [40]. Isolates of *A. baumannii, K. pneumoniae* and *P. aeruginosa* that are resistant to all, or almost all, commercially available antibiotics actually seem to be increasing worldwide [41]. In our population sample, *A. baumannii* and *K. pneumoniae* isolates included antibiotic-resistant and non-resistant strains, albeit in different proportions (39.3% vs. 18.2%, respectively), whereas all the isolated *Pseudomonas* spp. were found to be non-resistant.

In the Gram-positive group, *S. aureus* was the most prevalent bacterium with a total of 97 isolates, 13.4% of which were antibiotic-resistant, namely, methicillin-resistant *S. aureus*. In contrast, no coagulase-negative staphylococci were isolated. It is known that staphylococcal pneumonia is caused by *S. aureus*, which usually spreads to the lung through the blood from other infected sites, most often the skin. Though a common community pathogen, it is found twice as frequently in pneumonias occurring in hospitalized patients [42].

Among the other bacterial isolates detected in our HARI patients, there was also *Chryseobacterium gleum*, a rare but concerning device-associated Gram-negative organism that can cause pneumonia and urinary tract infections [43]. Risk factors include being neonate or immunocompromised, intensive care unit length of stay >21 days, broad-spectrum antibiotic exposure, indwelling devices and mechanical ventilation [44].

Finally, it is worth mentioning the relevant presence of fungal species, mainly *Candida* spp. and *Aspergillus* spp., in the respiratory specimens of our HARI patients. Opportunistic fungi such as *Candida* and *Aspergillus* rarely cause VAP, and mostly occur in immunocompromised patients [45,46]. However, many patients in ICUs have respiratory specimens positive for *Candida* without clinical and/or pathological evidence of invasive candidiasis, known as *Candida* colonization [47]. The detection of *Aspergillus* in airway secretions is not common in comparison with *Candida*, and its significance depends on the immune status of the patient. In severe immunosuppression such as neutropenia, organ transplantation, and corticosteroid therapy, the detection of *Aspergillus* in the airways suggests invasive aspergillosis, whereas in immunocompetent patients it typically represents colonization [48,49,50]. Although the clinical impact of airway colonization with these fungi is not clearly addressed, now it is obvious that, in immunocompetent patients under mechanical ventilation, the presence of *Candida* or *Aspergillus* in the respiratory tract is associated with worse outcomes of bacterial VAP and the lower survival of patients. This is greatly evident in the case of *Candida* colonization [45]. Thus, in critically ill patients, specific treatment should be considered if features of pulmonary infection are present and the above fungal species are isolated from respiratory secretions [47,48,49,50].

Our work has some limitations, among which include the lack of evaluation of the clinical symptoms, results of blood tests and instrumental investigations due to the methodology used in our research. However, the study provides a picture of the epidemiological situation of HARIs in the COVID-19 pre-pandemic period. In this regard, our results could contribute to explain, at least in part, the COVID-19 mortality rate observed in our country during the pandemic period. In fact, people who died from COVID-19 were mostly elderly, affected by chronic diseases, hospitalized in intensive care units and subjected to intubation and mechanical ventilation. Therefore, because of the need for hospitalization, patients with COVID-19 were exposed to additional risks linked to hospitalization, including the possibility of developing HAIs.

In conclusion, our study shows that acquiring respiratory infections following hospitalization is very frequent and that, especially, elderly patients, patients affected by chronic diseases, patients undergoing intubation/ventilation and patients with antibiotic-resistant microorganisms risk a more severe clinical outcome with significant mortality. The research methodology we used in this investigation, namely, the cross-referencing of demographic and clinical data obtained from hospital discharge forms with microbiological data extracted from the computer system of the microbiology unit, has proven to be effective. In fact, it has allowed us to perform a quick and accurate assessment of the burden, microbial etiology and clinical impact of HARIs at our institution in the period examined, as we had already experienced in a recent parallel study on viral respiratory infections [51].

The availability of fast and reliable tools for the constant monitoring of HAIs is essential to be able to keep up with the increase in microbial antibiotic resistance, which is also favored by the increase in chronic diseases, immunosuppression conditions, and aging of the general population. The international scientific community is currently working in this direction [52,53,54,55,56], being aware that only a thorough knowledge of local epidemiology and antibiotic resistance profiles can drive hospital institutions towards more successful prevention and treatment strategies.

## Figures and Tables

**Table 1:** Demographic and clinical characteristics of patients with HARI over a 12-month period.

Continuous Variables	Median (IQR)	Min	Max
Age (years)	0 (IQR 58–77)	18	93
Length of stay (days)	29 (16–51)	2	223
**Dichotomous Variables**		***n***	**%**
Gender	F	96	29.2
	M	233	70.8
Marital status	Married	271	82.4
	Single	58	17.6
Citizenship	Italian	302	91.8
	Non-Italian	27	8.2
Education level	Below high school	33	10
	Equal/over high school	296	90
Working activity	Unemployed/Retired	239	72.6
	Employed	90	27.4
Patients with microorganisms below or above the sample mean (mean 1.76 ± 0.96)	Below the sample mean (<2)	167	50.8
	Above the sample mean (≥2)	162	49.2
Patients with antibiotic resistant microorganisms (mean 1.09 ± 0.29)	At least 1 resistant organism	68	20.7
	2 resistant organisms	7	2.1
	None	254	77.2
Chronic diseases (dichotomous)	Absent	189	57.4
	Present	140	42.6
Death during hospitalization	No	212	64.4
	Yes	117	35.6
Major diagnostic category (MDC)	Medical	169	51.4
	Surgical	160	48.6
Intubation/ventilation	No	198	60.2
	Yes	131	39.8

**Table 2:** Distribution of microbiological isolates in respiratory specimens from patients with HARI.

Organism	Resistant ^		Non-Resistant ^		Total	
	N	% *	N	% *	N	% *
*Achromobacter*	0	0.0	3	0.6	3	0.5
*Acinetobacter baumannii*	24	29.3	37	7.4	61	10.5
*Aeromonas*	0	0.0	1	0.2	1	0.2
*Aspergillus spp.*	6	7.3	7	1.4	13	2.2
*Aureobasidium*	0	0.0	1	0.2	1	0.2
*Candida spp.*	0	0.0	92	18.4	92	15.8
*Chryseobacterium gleum*	0	0.0	4	0.8	4	0.7
*Citrobacter*	0	0.0	3	0.6	3	0.5
*Debaryomyces*	0	0.0	1	0.2	1	0.2
*Delftia*	0	0.0	2	0.4	2	0.3
*Enterobacter spp.*	8	9.8	23	4.6	31	5.3
*Enterococcus spp.*	4	4.9	24	4.8	28	4.8
*Escherichia coli*	5	6.1	25	5.0	30	5.2
*Hafnia*	0	0.0	1	0.2	1	0.2
*Haemophilus influenzae*	0	0.0	3	0.6	3	0.5
*Klebsiella pneumoniae*	14	17.1	63	12.6	77	13.3
*Morganella morganii*	2	2.4	0	0.0	2	0.3
*Mycobacterium tuberculosis*	0	0.0	2	0.4	2	0.3
*Proteus spp.*	0	0.0	13	2.6	13	2.2
*Providencia*	0	0.0	2	0.4	2	0.3
*Pseudomonas spp.*	0	0.0	72	14.4	72	12.4
*Raoultella*	0	0.0	4	0.8	4	0.7
*Rothia*	0	0.0	1	0.2	1	0.2
*Serratia marcescens*	1	1.2	6	1.2	7	1.2
*Sfingobacterium*	0	0.0	1	0.2	1	0.2
*Staphylococcus aureus*	13	15.9	84	16.8	97	16.7
*Stenothropomonas maltophilia*	3	3.7	12	2.4	15	2.6
*Streptococcus pyogenes*	0	0.0	6	1.2	6	1.0
*Streptococcus pneumoniae*	2	2.4	6	1.2	8	1.4
**Total**	**82**	**100.0**	**499**	**100.0**	**581**	**100.0**

* Percentage calculated on the total of the specific field (resistant, non-resistant and total). ^ Antibiotic-resistant and non-resistant organisms.

**Table 3:** Crude and adjusted OR (and 95% CI) of death, according to relevant variables.

Variables	Reference Category	Univariable Analysis				Multivariable Analysis			
		OR	IC 95%		*p* *	OR ADJ *	IC 95%		*p* **
**Age (years)**		N/A	N/A	N/A	***0.060 ^***	**1.020**	**1.000**	**1.040**	***0.050***
Length of stay (days)		N/A	N/A	N/A	*0.687 ^*				
Gender (m/f)	*M*	1.149	0.696	1.896	*0.588*				
Civil status (married/single)	*Married*	1.730	0.915	3.272	***0.092***	0.752	0.345	1.640	*0.473*
Citizenships (Italian/non-Italian)	*Italian*	1.341	0.568	3.166	*0.503*				
Education level (high/low)	*Low*	0.962	0.455	2.033	*0.919*				
Work activity	*Unemployed*	1.000	0.603	1.660	*0.999*				
**Major diagnostic category (MDC) (medical/surgical)**	***Surgical***	1.539	0.978	2.424	***0.063***	**2.606**	**1.295**	**5.241**	***0.007***
Patients with antibiotic-resistant organisms	*Yes*	2.824	1.666	4.789	***0.0001***	1.230	0.616	2.458	*0.558*
**Patients with microorganisms above the sample mean (≥2)**	***Yes***	3.428	2.126	5.527	***0.0001***	**2.500**	**1.424**	**4.415**	***0.001***
**Chronic diseases**	***Yes***	1.645	1.043	2.596	***0.032***	**1.753**	**1.010**	**3.043**	***0.046***
**Intubation/ventilation**	***Yes***	7.794	4.687	12.959	***0.0001***	**12.274**	**5.960**	**25.278**	***0.0001***

Dependent variable: death; ^ Mann–Whitney U test (for continuous variables); * in bold *p* values ≤ 0.10 at univariable level; ** in bold *p* values ≤0.05 at multivariable level; empty cells: variable not included in the model. N/A: not applicable.

**Table 4:** Crude and adjusted ORs (and 95% CI) of intubation/ventilation, according to relevant variables.

Variables	Reference Category	Univariable Analysis				Multivariable Analysis			
		OR	IC 95%		*p* *	OR ADJ *	IC 95%		*p* **
**Age (years)**		N/A	N/A	N/A	0.12 ^				
Length of stay (days)		N/A	N/A	N/A	**0.0001 ^**	1.009	0.999	1.019	0.077
Death	***Yes***	7.794	4.687	12.959	**0.0001**	**12.140**	**6.004**	**24.548**	**0.0001**
Gender (m/f)	***M***	1.683	1.018	2.785	**0.043**	**2.062**	**1.023**	**4.156**	**0.043**
Civil status (married/single)	*Married*	1.204	0.669	2.167	0.536				
Citizenship (Italian/non-Italian)	*Italian*	0.768	0.321	1.696	0.474				
Education level (low/high)	*Low*	0.629	0.289	1.369	0.242				
Work activity (employed/unemployed)	*Unemployed*	1.055	0.642	1.733	0.833				
**Main diagnostic category (MDC) (medical/surgical)**	***Surgical***	7.932	4.769	13.192	**0.0001**	**8.894**	**4.363**	**18.128**	**0.0001**
**Patients with antibiotic-resistant organisms**	***Yes***	6.441	3.614	11.480	**0.0001**	**2.977**	**1.440**	**6.156**	**0.003**
Patients with microorganisms above the sample mean (≥2)	*Yes*	3.243	2.042	5.150	**0.0001**	1.264	0.686	2.330	0.453
Chronic diseases	*Yes*	0.823	0.525	1.288	0.394				

Dependent variable: ventilation/intubation; ^ Mann–Whitney U test (for continuous variables). * in bold *p* values ≤ 0.10 at univariable level; ** in bold *p* values ≤ 0.05 at multivariable level; empty cells: variable not included in the model. N/A: not applicable.

**Table 5:** Multivariable linear regression for the dependent variable of length of stay (in days).

Variable	B	*p* *	Confidence Interval for B 95%	
			Lower Limit	Upper Limit
**Age (years)**	**0.037**	0.716	−0.163	0.237
Death	**−11.053**	**0.0002**	**−16.824**	**−5.283**
Male gender	0.930	0.725	−4.268	6.129
Civil status—married	−3.775	0.266	−10.436	2.886
Citizenship—Italian	7.488	0.113	−1.787	16.763
Educational level—high	−6.510	0.108	−14.458	1.437
Work activity—employed	−0.520	0.870	−6.780	5.739
MDC—surgical	**14.214**	**0.0001**	**8.795**	**19.633**
Patients with antibiotic-resistant organisms	**7.810**	**0.015**	**1.513**	**14.106**
Patients with microorganisms above the sample mean (≥2)	**9.475**	**0.0003**	**4.405**	**14.546**
Patients with chronic diseases—	−0.511	0.834	−5.312	4.291
Patients with intubation/mechanical ventilation	**7.542**	**0.022**	**1.108**	**13.976**

Dependent variable: length of stay (days); * in bold *p* values ≤ 0.05.

## Data Availability

Restrictions apply to the availability of these data. Data was obtained from third party (Fondazione PTV, Rome) and are eventually available with the permission of third party.

## References

[1] Monegro A.F., Muppidi V., Regunath H. (2022). Hospital Acquired Infections.

[2] Suetens C., Latour K., Kärki T., Ricchizzi E., Kinross P., Moro M.L., Jans B., Hopkins S., Hansen S., Lyytikäinen O. (2018). Prevalence of healthcare-associated infections, estimated incidence and composite antimicrobial resistance index in acute care hospitals and long-term care facilities: Results from two European point prevalence surveys, 2016 to 2017. Euro Surveill..

[3] Fair R.J., Tor Y. (2014). Antibiotics and bacterial resistance in the 21st century. Perspect. Medicin. Chem..

[4] Voidazan S., Albu S., Toth R., Grigorescu B., Rachita A., Moldovan I. (2020). Healthcare Associated Infections-A New Pathology in Medical Practice?. Int. J. Environ. Res. Public Health.

[5] GBD 2017 Causes of Death Collaborators (2018). Global, regional, and national age-sex-specific mortality for 282 causes of death in 195 countries and territories, 1980–2017: A systematic analysis for the Global Burden of Disease Study 2017. Lancet.

[6] Barbier F., Andremont A., Wolff M., Bouadma L. (2013). Hospital-acquired pneumonia and ventilator-associated pneumonia: Recent advances in epidemiology and management. Curr. Opin. Pulm. Med..

[7] Kalil A.C., Metersky M.L., Klompas M., Muscedere J., Sweeney D.A., Palmer L.B., Napolitano L.M., O’Grady N.P., Bartlett J.G., Carratalà J. (2016). Management of adults with hospital-acquired and ventilator-associated pneumonia: 2016 clinical practice guidelines by the Infectious Diseases Society of America and the American Thoracic Society. Clin. Infect. Dis..

[8] Tamma P.D., Goodman K.E., Harris A.D., Tekle T., Roberts A., Taiwo A., Simner P.J. (2017). Comparing the outcomes of patients with carbapenemaseproducing and non-carbapenemase-producing carbapenem-resistant *Enterobacteriaceae* bacteremia. Clin. Infect. Dis..

[9] Dajko M., Poscia A., Posteraro B., Speziale D., Volpe M., Mancinelli S., Ricciardi W., de Waure C. (2020). Microbiological ascertainment in patients with pneumonia: The experience of a teaching hospital in Rome. Ann. Ist. Super. Sanità.

[10] Carroll K.C. (2002). Laboratory diagnosis of lower respiratory tract infections: Controversy and conundrums. J. Clin. Microbiol..

[11] Jean S.S., Chang Y.C., Lin W.C., Lee W.S., Hsueh P.R., Hsu C.W. (2020). Epidemiology, Treatment, and Prevention of Nosocomial Bacterial Pneumonia. J. Clin. Med..

[12] Torres A., Niederman M.S., Chastre J., Ewig S., Fernandez-Vandellos P., Hanberger H., Kollef M., Li Bassi G., Luna C.M., Martin-Loeches I. (2017). International ERS/ESICM/ESCMID/ALAT guidelines for the management of hospital-acquired pneumonia and ventilator-associated pneumonia: Guidelines for the management of hospital-acquired pneumonia (HAP)/ventilator-associated pneumonia (VAP) of the European Respiratory Society (ERS), European Society of Intensive Care Medicine (ESICM), European Society of Clinical Microbiology and Infectious Diseases (ESCMID) and Asociación Latinoamericana del Tórax (ALAT). Eur. Respir. J..

[13] Weber D., Rutala W., Sickbert-Bennet E., Samsa G., Brown V., Niederman M. (2007). Microbiology of ventilator-associated pneumonia compared with that of hospital-acquired pneumonia. Infect. Control. Hosp. Epidemiol..

[14] Khan H.A., Ahmad A., Mehboob R. (2015). Nosocomial infections and their control strategies. Asian Pac. J. Trop. Biomed..

[15] Dingle T.C., Butler-Wu S.M. (2013). Maldi-tof mass spectrometry for microorganism identification. Clin. Lab. Med..

[16] Favaro M., Sarti M., Fontana C. (2014). Multiplex Real-Time PCR probe-based for identification of strains producing: OXA48, VIM, KPC and NDM. World J. Microbiol. Biotechnol..

[17] Magiorakos A.P., Srinivasan A., Carey R.B., Carmeli Y., Falagas M.E., Giske C.G., Harbarth S., Hindler J.F., Kahlmeter G., Olsson-Liljequist B. (2012). Multidrug-resistant, extensively drug-resistant and pandrug-resistant bacteria: An international expert proposal for interim standard definitions for acquired resistance. Clin. Microbiol. Infect..

[18] Zingg W., Holmes A., Dettenkofer M., Goetting T., Secci F., Clack L., Allegranzi B., Magiorakos A.P., Pittet D., Systematic review and evidence-based guidance on organization of hospital infection control programmes (SIGHT) study group (2015). Hospital organisation, management, and structure for prevention of health-care-associated infection: A systematic review and expert consensus. Lancet Infect. Dis..

[19] Secondo Studio di Prevalenza Italiano sulle Infezioni Correlate all’Assistenza e Sull’uso di Antibiotici Negli Ospedali per Acuti—Protocollo ECDC, 2018. Dipartimento Scienze della Salute Pubblica e Pediatriche, Università di Torino. https://www.salute.gov.it/imgs/C_17_pubblicazioni_2791_allegato.pdf.

[20] Fried L.P., Ferrucci L., Darer J., Williamson J.D., Anderson G. (2004). Untangling the concepts of disability, frailty, and comorbidity: Implications for improved targeting and care. J. Gerontol. A. Biol. Sci. Med. Sci..

[21] Offner P.J., Moore E.E., Biffl W.L. (1999). Male gender is a risk factor for major infections after surgery. Arch. Surg.

[22] McClelland E.E., Smith J.M. (2011). Gender Specific Differences in the Immune Response to Infection. Arch. Immunol. Ther. Exp..

[23] Bassetti M., Taramasso L., Giacobbe D.R., Pelosi P. (2012). Management of ventilator-associated pneumonia: Epidemiology, diagnosis and antimicrobial therapy. Expert Rev. Anti. Infect. Ther..

[24] Magill S.S., O’Leary E., Janelle S.J., Thompson D.L., Dumyati G., Nadle J., Wilson L.E., Kainer M.A., Lynfield R., Greissman S. (2018). Emerging infections program hospital prevalence survey team. Changes in prevalence of health care-associated infections in U.S. hospitals. N. Engl. J. Med..

[25] Delle Rose D., Pezzotti P., Fortunato E., Sordillo P., Gini S., Boros S., Meledandri M., Gallo M.T., Prignano G., Caccese R. (2016). Clinical predictors and microbiology of ventilator-associated pneumonia in the intensive care unit: A retrospective analysis in six Italian hospitals. Eur. J. Clin. Microbiol. Infect. Dis..

[26] Ferrer M., Torres A. (2018). Epidemiology of ICU-acquired pneumonia. Curr. Opin. Crit. Care.

[27] Ranzani O.T., De Pascale G., Park M. (2018). Diagnosis of non ventilated hospital-acquired pneumonia: How much do we know?. Curr. Opin. Crit. Care.

[28] Giuliano K.K., Baker D., Quinn B. (2018). The epidemiology of nonventilator hospital-acquired pneumonia in the United States. Am. J. Infect. Control.

[29] Bartlett J.G., O’Keefe P., Tally F.P., Louie T.J., Gorbach S.L. (1986). Bacteriology of hospital-acquired pneumonia. Arch. Intern. Med..

[30] Peters B.M., Jabra-Rizk M.A., O’May G.A., Costerton J.W., Shirtliff M.E. (2012). Polymicrobial interactions: Impact on pathogenesis and human disease. Clin. Microbiol. Rev..

[31] Lode H., Schaberg T., Raffenberg M., Mauch H. (1995). Lower respiratory tract infections in the intensive care unit: Consequences of antibiotic resistance for choice of antibiotic. Microb. Drug Resist..

[32] Celis R., Torres A., Gatell J.M., Almela M., Rodríguez-Roisin R., Agustí-Vidal A. (1988). Nosocomial pneumonia. A multivariate analysis of risk and prognosis. Chest.

[33] Pittet D., Li N., Wenzel R.P. (1993). Association of secondary and polymicrobial nosocomial bloodstream infections with higher mortality. Eur. J. Clin. Microbiol. Infect. Dis..

[34] Zheng C., Zhang S., Chen Q., Zhong L., Huang T., Zhang X., Zhang K., Zhou H., Cai J., Du L. (2020). Clinical characteristics and risk factors of polymicrobial *Staphylococcus aureus* bloodstream infections. Antimicrob. Resist. Infect. Control.

[35] de Kraker M.E., Davey P.G., Grundmann H., BURDEN study group (2011). Mortality and hospital stay associated with resistant *Staphylococcus aureus* and *Escherichia coli* bacteremia: Estimating the burden of antibiotic resistance in Europe. PLoS Med..

[36] Mauldin P.D., Salgado C.D., Hansen I.S., Durup D.T., Bosso J.A. (2010). Attributable hospital cost and length of stay associated with health care-associated infections caused by antibiotic-resistant gram-negative bacteria. Antimicrob. Agents. Chemother..

[37] Paterson D.L. (2006). The epidemiological profile of infections with multidrug-resistant *Pseudomonas aeruginosa* and *Acinetobacter* species. Clin. Infect. Dis..

[38] American Thoracic Society, Infectious Diseases Society of America (2005). Guidelines for the management of adults with hospital-acquired, ventilator-associated, and healthcare-associated pneumonia. Am. J. Respir. Crit. Care. Med.

[39] Seifert H., Dijkshoorn L., Gerner-Smidt P., Pelzer N., Tjernberg I., Vaneechoutte M. (1997). Distribution of Acinetobacter species on human skin: Comparison of phenotypic and genotypic identification methods. J. Clin. Microbiol.

[40] Paczosa M.K., Mecsas J. (2016). *Klebsiella pneumoniae*: Going on the offense with a strong defense. Microbiol. Mol. Biol. Rev..

[41] Biedenbach D.J., Giao P.T., Hung Van P., Su Minh Tuyet N., Thi Thanh Nga T., Phuong D.M., Vu Trung N., Badal R.E. (2016). Antimicrobial-resistant *Pseudomonas aeruginosa* and *Acinetobacter baumannii* from patients with hospital-acquired or ventilator-associated pneumonia in Vietnam. Clin. Ther..

[42] Farver C.F., Zander D.S. (2009). Molecular Basis of Pulmonary Disease. Mol. Pathol..

[43] Tsouvalas C.P., Mousa G., Lee A.H., Philip J.A., Levine D. (2020). Chryseobacterium gleum isolation from respiratory culture following community-acquired pneumonia. Am. J. Case Rep..

[44] Amisha F., Fugere T., Caceres J., Rico Crescencio J.C., Falls N. (2021). Chryseobacterium gleum Causing Healthcare-Associated Pneumonia in an Adult Male with Diffuse Large B Cell Lymphoma. Cureus.

[45] Khorvash F., Abbasi S., Yaran M., Abdi F., Ataei B., Fereidooni F., Hoseini S.G., Ahmadi-Ahvaz N., Parsazadeh M., Haghi F. (2014). Molecular detection of *Candida* spp. and *Aspergillus fumigatus* in bronchoalveolar lavage fluid of patients with ventilator-associated pneumonia. J. Res. Med. Sci..

[46] Garnacho-Montero J., Amaya-Villar R., Ortiz-Leyba C., León C., Alvarez-Lerma F., Nolla-Salas J., Iruretagoyena J.R., Barcenilla F. (2005). Isolation of *Aspergillus* spp. from the respiratory tract in critically ill patients: Risk factors, clinical presentation and outcome. Crit. Care.

[47] Meersseman W., Lagrou K., Spriet I., Maertens J., Verbeken E., Peetermans W.E., Van Wijngaerden E. (2009). Significance of the isolation of *Candida* species from airway samples in critically ill patients: A prospective autopsy study. Intensive Care Med..

[48] Garnacho-Montero J., Olaechea P., Alvarez-Lerma F., Alvarez-Rocha L., Blanquer J., Galván B., Rodriguez A., Zaragoza R., Aguado J.M., Mensa J. (2013). Epidemiology, diagnosis and treatment of fungal respiratory infections in the critically ill patient. Rev. Esp. Quimioter..

[49] Ricard J.D., Roux D. (2012). *Candida* colonization in ventilated ICU patients: No longer a bystander!. Intensive Care Med..

[50] Humphreys H., Johnson E.M., Warnock D.W., Willatts S.M., Winter R.J., Speller D.C. (1991). An outbreak of aspergillosis in a general ITU. J. Hosp. Infect.

[51] Ciotti M., Maurici M., Santoro V., Coppola L., Sarmati L., De Carolis G., De Filippis P., Pica F. (2020). Viruses of respiratory tract: An observational retrospective study on hospitalized patients in Rome, Italy. Microorg..

[52] Capozzi C., Maurici M., Panà A. (2019). Antimicrobico resistenza: È crisi globale, “un lento tsunami” [Antimicrobial resistance: It is a global crisis, “a slow tsunami”]. Ig. Sanita. Pubblica.

[53] Paterson David L. (2006). The Role of Antimicrobial management programs in optimizing antibiotic prescribing within hospitals. Clin. Infect. Dis..

[54] Fontana C., Angeletti S., Mirandola W., Cella E., Alessia L., Zehender G., Favaro M., Leoni D., Rose D.D., Gherardi G. (2020). Whole genome sequencing of carbapenem-resistant *Klebsiella pneumoniae*: Evolutionary analysis for outbreak investigation. Future Microbiol..

[55] Plachouras D., Kärki T., Hansen S., Hopkins S., Lyytikäinen O., Moro M.L., Reilly J., Zarb P., Zingg W., Kinross P. (2018). Antimicrobial use in European acute care hospitals: Results from the second point prevalence survey (PPS) of healthcare-associated infections and antimicrobial use, 2016 to 2017. Euro Surveill..

[56] Tacconelli E., Cataldo M.A., Dancer S.J., De Angelis G., Falcone M., Frank U., Kahlmeter G., Pan A., Petrosillo N., Rodríguez-Baño J. (2014). ESCMID guidelines for the management of the infection control measures to reduce transmission of multidrug-resistant Gram-negative bacteria in hospitalized patients. Clin. Microbiol. Infect..

